# Knowledge, attitudes, and practices of people living in artisanal mining areas on water pollution in Siguiri, Guinea, 2023

**DOI:** 10.3389/fpubh.2025.1482032

**Published:** 2025-04-10

**Authors:** Aly Badara Toure, Mariama Sadjo Diallo, Sidikiba Sidibe, Salifou Talassone Bangoura, Fassou Mathias Grovogui, Maladho Diaby, Mohamed Aly Bangoura, Yamoussa Youla, Mory Kourouma, Alexandre Delamou

**Affiliations:** ^1^African Center of Excellence for the Prevention and Control of Communicable Diseases (CEA-PCMT), University Gamal Abdel Nasser of Conakry, Conakry, Guinea; ^2^Département de Santé Publique, Université Gamal Abdel Nasser de Conakry, Conakry, Guinea; ^3^Laboratoire de Biologie Moléculaire Nestor Bangoura/Hélène Labrousse, CTA Hôpital National Donka, Conakry, Guinea; ^4^Centre de Recherche et de Formation en Infectiologie de Guinée (CERFIG), Campus Hadja Mafory Bangoura, Donka, Conakry, Guinea; ^5^Centre National de Formation et de Recherche en Santé Rurale de Maferinyah (CNSFRSM), Forécariah, Guinea; ^6^Département des Sciences Pharmaceutiques et Biologiques de l'Université Gamal Abdel Nasser de Conakry, Conakry, Guinea; ^7^Centre Emergent des Mines et Sociétés de Boké (CEMS), Institut Supérieur des Mines et, Géologies de Boké, Guinea

**Keywords:** attitude, artisanal mining, knowledge, practice, Siguiri, water pollution

## Abstract

**Introduction:**

Water pollution is a major public health issue, especially in mining areas where artisanal mining activities are prevalent. The objective of this study was to analyze the knowledge, attitudes, and practices (KAP) of the population regarding water pollution in the mining areas of Siguiri, Guinea.

**Methods:**

A cross-sectional study was conducted from May 15 to June 15, 2023, on the population of Doko, Siguiri. Data were collected using a structured questionnaire that assessed knowledge, attitudes, and practices related to water pollution in artisanal mining areas. Logistic regression was used to analyze factors associated with KAP.

**Results:**

The survey included 501 respondents. Good knowledge of water pollution was observed in 53% of respondents, while 52% exhibited a positive attitude towards water pollution in artisanal mining area. Good practices were reported by 81% of respondents. The multivariate analysis showed that being educated (ORa: 2.13; 95% CI: 1.39; 3.29) and being a foreigner (ORa: 1.88; 95% CI: 1.04; 3.51) significantly associated with positive attitudes towards water pollution in artisanal mining area. Indeed, being single (aOR: 1.84; 95% CI: 1.10; 3.14), having good knowledge of water pollution (aOR: 2.51; 95% CI: 1.47; 4.36) and lack of lifestyle (tobacco and alcohol) (aOR: 2.51; 95% CI: 1.02; 5.97) were significantly associated with good practices.

**Conclusion:**

This study revealed moderate knowledge, positive attitudes and adequate practices regarding water pollution in Siguiri’s artisanal mining areas. However, significant gaps remain, including a lack of awareness of the risks associated with prolonged exposure to heavy metals. She advocates an integrated approach combining education, awareness-raising and technical support, accompanied by concrete solutions such as water treatment and the strengthening of community initiatives, in order to convert this knowledge into sustainable behavior.

## 1 Introduction

Water plays an essential role in the survival and health of living beings, as well as in the socio-economic development of communities. When it is intended for consumption, it must be free from any contamination, whether of chemical or biological origin, likely to be harmful to human health (1). Water pollution is therefore a major public health problem. A number of factors, both natural and man-made, are known to have a negative impact on water quality. These include agricultural activities, the production of industrial and municipal waste, construction, and mining operations (2).

Artisanal and small-scale gold mining is a growing activity in many countries around the world (3). It is practiced by more than 40 million people in 120 countries, providing a livelihood for between 80 and 150 million people in low- and middle-income countries (4). Despite its important role in developing the informal economy within communities, artisanal mining also has environmental disadvantages. It generates environmental stress that poses a serious threat to water resources and human health (5).

In mining, water is of vital importance not only for the mining activity but also for the consumption and hygiene of the community (6). However, water bodies are particularly vulnerable to contamination by heavy metals and micro-organisms because of the poor hygiene often observed in these areas (7, 8).

In Guinea, artisanal mining has been practiced for over a century in regions rich in gold and diamond (9). It is carried out in several prefectures, and the miners involved are estimated at over 200,000, with 15% of these miners coming from neighboring ECOWAS countries. For local residents, this is a seasonal activity, carried out at the end of the agricultural season (10).

The prefecture of Siguiri is an area where small-scale gold mining is carried out industrially by the Société Aurifère de Guinee (SAG) and artisanally by the local population. Mining is carried out through a dense network of shafts built along watercourses and in forest galleries (11). On the other hand, access to drinking water is a major public health and development issue worldwide. That’s why water pollution is a priority in the Sustainable Development Goals (SDGs), particularly SDG 6, which aims to guarantee access to drinking water for all (12). According to the 2018 Guinea Demographic and Health Survey (EDS V) (13), 79% of households consume water from improved sources, compared with 30% of the rural population who use unimproved sources, and 71% of the population who do not use any water treatment.

Inadequate knowledge, attitudes and practices regarding the management of water bodies can have a negative impact on water quality from the source to the place of use (14, 15). Several studies have shown the positive effects of good knowledge of water pollution and its consequences for good practice in preventing the phenomenon in sub-Saharan Africa (6, 16, 17). However, few studies have looked at people’s knowledge, attitudes and practices regarding water pollution in artisanal mining areas.

In Guinea, several studies have been carried out on artisanal mining (7, 11, 18, 19). However, none of these studies has assessed the knowledge, attitudes and practices of the population in relation to water pollution.

The findings of this study will help to raise public awareness of water pollution, support the design of water pollution prevention projects, and raise awareness in artisanal mining areas. These efforts are also part of a global perspective, supporting international initiatives to reduce water pollution and protect vulnerable ecosystems, while promoting sustainable practices in artisanal mining areas.

The aim of this study was to assess the population’s knowledge, attitudes and practices in relation to water pollution at artisanal mining sites in Siguiri.

## 2 Methods

### 2.1 Study design and period

This was a cross-sectional study conducted from 15 May to 15 June 2023 among communities living in the gold mining areas of the Doko sub-prefecture in the Siguiri health district in Guinea.

### 2.2 Study area

The Republic of Guinea is a West African country with a surface area of 245,857 km^2^ with an estimated 14 million inhabitants in 2022. Around 76% of the population lived in rural areas in 2017 and the literacy rate was 40% in 2018 (20). It has significant mining potential, with reserves of bauxite, iron, gold, diamonds, etc. …. The mining sector is a pillar of the Guinean economy, accounting for more than 85% of exports and providing around 35% of gross domestic product (GDP) in 2020. In 2019, 44% of Guineans were living below the national poverty line and the economy was largely informal (21).

**Figure 1:**
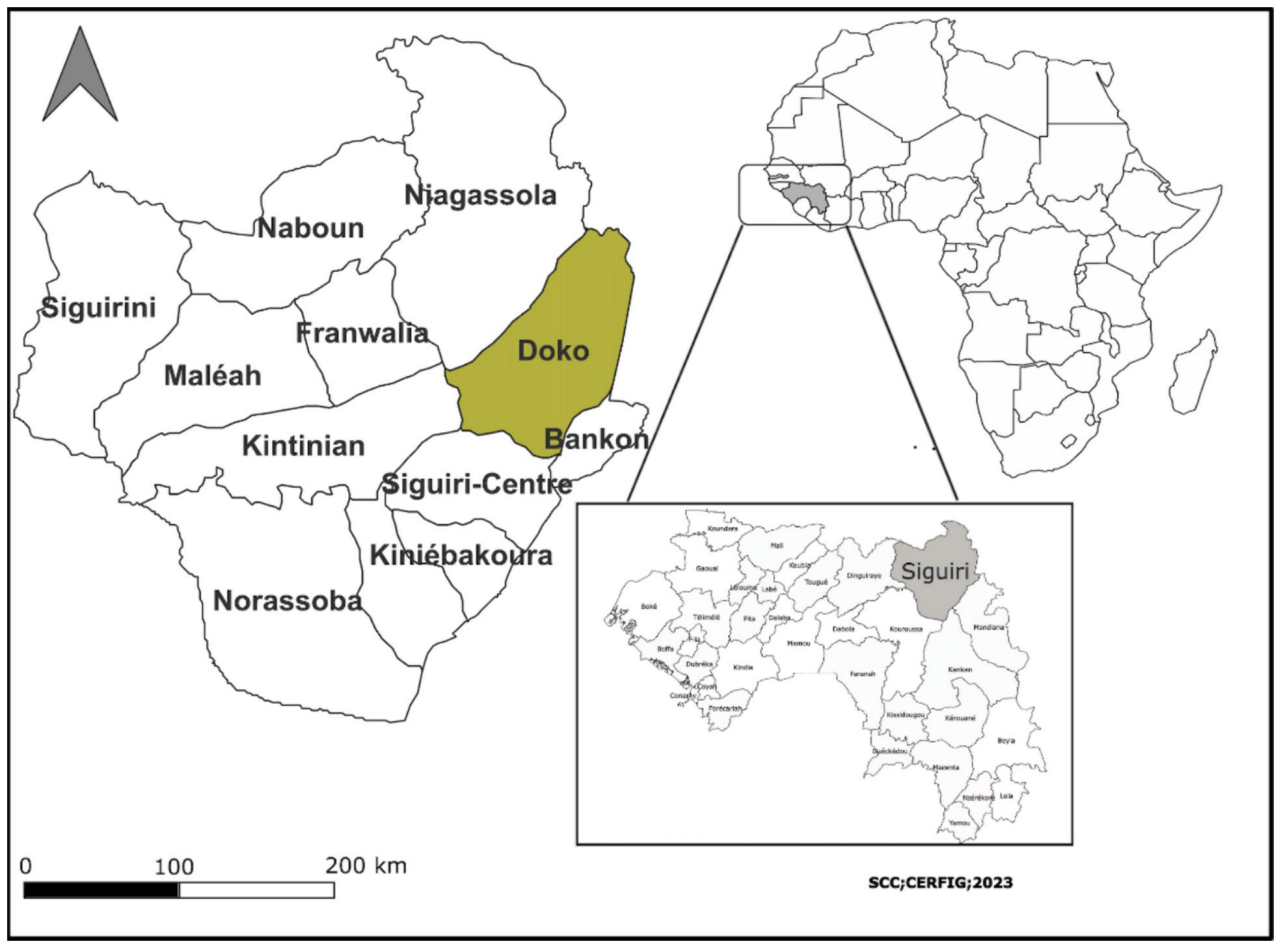
Map of the Siguiri prefecture and its sub-prefectures.

Siguiri is a prefecture located in the extreme north-east of the Republic of Guinea (Figure 1), about 850 km by road from the capital Conakry (11). It has numerous artisanal gold mining sites, particularly in almost all the localities in the prefecture. The region’s reputation for gold mining is recognised worldwide, and the mining and trade of the mineral have gone on for several centuries, even if modern techniques have transformed it considerably (22). This study was carried out in three sub-prefectures with artisanal gold mining sites (Oudoula Damafè, Doko centre, Kodiarani 1) due to the high number of mine workers according to the National Action Plan for Artisanal and Small-scale Gold Mining (EMAPE) (10).

### 2.3 Study population

The study population consisted of all people aged 18 and over who consented to participate in the study without any inducments.

### 2.4 Sample size and sampling

The minimum sample size was calculated using the Schwartz formula (23). The prevalence used was the proportion of Guinean households utilizing at least one basic drinking water service (access to an improved source) in rural areas (*p* = 48.7%) (12). The sample size obtained was 357; after correcting for a non-response rate of 10%, we obtained 501.

Sampling was conducted using random sampling technique in order to obtain a representative sample of the population. Households were selected from the Siguiri household enumeration database. In each household selected, we counted all the people eligible for our study. We then interviewed the head of the household or his/her spouse if he or she was unavailable. If both were absent or unavailable, we interviewed the oldest resident present. If the selected household was locked or if no eligible participant was present at the time of the visit, the household was revisited the following day. If no member was available, then another household was selected to replace it from a replacement list that was drawn up. All the interviews were conducted during the day.

### 2.5 Data collection method

Individual face-to-face interviews were conducted using an anonymous, standardized questionnaire. The questionnaire was entered into the Kobo collect software and then loaded onto tablets to facilitate data entry. It was pre-tested and validated before data collection began. Data collectors were recruited and trained on the purpose and methods of the study to ensure consistency and reliability of data collection. The principal investigator supervised the entire data collection and management.

### 2.6 Study variables

#### 2.6.1 Outcome variable

##### 2.6.1.1 Attitudes

Practices were defined on the basis of 4 close-ended questions on the source of drinking water, the treatment of drinking water at home, the type of container used for storing water at home and the use of latrines. For each question, a response was considered ‘correct’ if it aligned with scientifically validated practices or demonstrated a positive attitude toward recommended behaviors (e.g., agreeing that boiling water eliminates harmful pathogens). Each correct answer had a value of “1” and the wrong answer or do not know had a value of “0.” Scores were added together to give a total score ranging from 0 to 4 points. Participants with a score equal to the total score were considered to have good practice. Those with a score below the total score were considered to have poor practice.

##### 2.6.1.2 Practices

Practices were defined on the basis of 4 close-ended questions on the source of drinking water, the treatment of drinking water at home, the type of container used for storing water at home and the use of latrines. Each correct answer had a value of “1” and the wrong answer or do not know had a value of “0.” Scores were added together to give a total score ranging from 0 to 4 points. Participants with a score equal to the total score were considered to have good practice. Those with a score below the total score were considered to have poor practice.

#### 2.6.2 Independent variables

##### 2.6.2.1 Knowledge

Knowledge was evaluated on the basis of 21 close-ended questions with sub-questions on contamination of drinking water by metals and microorganisms, health problems encountered, having information/education on drinking water quality, the reason for boiling water in a covered container, and having a latrine. Each correct answer had a value of “1” and the wrong answer or do not know had a value of “0.” Scores were added together to give a total score ranging from 0 to 21 points. Participants with a score above the mean score were considered to have good knowledge. Those with a score below or equal to the average score were considered to have poor knowledge.

Socio-demographic characteristics included: sex (male and female), age group (< 30; 30–45; >45 years), marital status (married and single), occupation (mining worker and non-mining worker), instruction level (unschooled and schooled), origin (foreigner and Guinean), lifestyle (tobacco and alcohol), religion (christian, fetishist and muslim).

### 2.7 Analysis

The data collected were analyzed using R Studio software (4.2.3). Descriptive statistics were produced. Qualitative variables were presented as frequencies and percentages, and quantitative variables as means and standard deviations. For comparison of categorical variables, a chi 2 test was performed. Factors associated with participants’ attitudes and practices were then analyzed using logistic regression. Variables were included in the final model after assessing for collinearity between independent variables. A mean–variance inflation factor (VIF) score of less than four is tolerated. The measures of association were estimated by odds ratios (ORs) with their 95% confidence intervals (CIs). All *p*-value values <0.05 were considered statistically significant.

## 3 Results

Overall, 501 individuals participated in the survey. The mean age of participants was 32.20 ± 10.28 years, and people aged below 30 years represented more than half of the sample (n = 259; 52%). Almost 74% were men, and 63% were married at the time of the survey. At least two out of three participants (68%) were not in school, and 42% reported being mineworkers (Table 1).

**Table 1:** Socio-demographic characteristics of respondents in the Doko mining area, Siguiri district, Guinea, June 2023 (*n* = 501).

Variables	Frequency	Percentage
Sex		
Female	129	26
Male	372	74
Age mean (SD) (years)	32 (±10)	
Age_group		
<30	259	52
30–45	194	39
>45	48	19
Marital status		
Married	317	63
Single	184	37
Occupation		
Mining worker	210	42
Non-mining worker	291	58
Education level		
Unschooled	339	68
Schooled	162	32
Origin		
Foreigner	55	11
Guinean	446	89
Lifestyle		
Alcohol	37	7
None	310	62
Tobacco	154	31
Religion		
Christian	24	5
Others	9	2
Muslim	468	93

Table 2 shows respondents’ level of knowledge about water pollution in artisanal mining areas. Overall, 53% of respondents had a good knowledge of water pollution. The majority (n = 476; 95%) knew that artisanal mining was a source of water pollution. Exposure to heavy metals and microbial germs could also occur via drinking water, according to 78 and 77% of respondents, respectively. About 96% said that ingesting germs could lead to diarrheal diseases. However, more than two-thirds of respondents (n = 334; 67%) did not know that ingesting large quantities of heavy metals could lead to cancer.

**Table 2:** Respondents’ knowledge of water pollution, Siguiri mining areas, Guinea, June 2023 (*n* = 501).

Variables	Frequency	Percent
Can water be contaminated?		
No/Do not know	15	5
Yes	476	95
Is household waste a source of pollution?		
No/Do not know	183	36
Yes	318	63
Are you aware that artisanal mining is a source of pollution?		
No/Do not know	25	5
Yes	476	95
Is leakage of heavy metals a source of pollution?		
No/Do not know	68	14
Yes	433	86
Did you know that excrement in nature is a source of pollution?		
No Do not know	56	11
Yes	445	89
Did you know that connecting latrines to the water supply is a source of pollution?		
No/Do not know	106	21
Yes	395	79
Does drinking water contain contaminants such as heavy metals?		
No/Do not know	101	21
Yes	400	79
Does drinking water contain contaminants such as germs?		
No/Do not know	107	21
Yes	396	79
Can exposure to heavy metals occur via drinking water?		
No/Do not know	111	22
Yes	390	78
Can exposure to microbial germs occur via drinking water?		
No/Do not know	115	23
Yes	386	77
Can heavy metals build up in the body?		
No/Do not know	111	22
Yes	390	78
Can ingesting a large amount of metals lead to cancer?		
No/Do not know	85	17
Yes	416	83
Can germs accumulate in the body?		
No/Do not know	334	67
Yes	167	33
Can the ingestion of many germs lead to diarrheal disease?		
No/Do not know	22	4
Yes	479	96
Did you know that polluted water is not good for drinking?		
No/Do not know	31	7
Yes	468	93
Does water from rivers, wells or boreholes need to be treated before drinking?		
No/Do not know	47	35
Yes	325	65
What are the consequences of liquid waste?		
Exposure to disease	396	79
Does not expose to disease	105	21
Can unsafe water cause diarrheal disease?		
No/Do not know	21	5
Yes	475	95
Have you ever received education/information about drinking water quality?		
No	249	50
Yes	252	50
The purpose of using a covered container to boil drinking water is to:		
Reduce contamination	463	92
Reduce boiling time	26	5
Both	12	3
Are latrines essential and compulsory for every household?		
No	160	32
Yes	341	68
Total average score (SD)	14,47 (± 1,21)	
Poor knowledge (≤14 points)	235	47
Good knowledge (>14 points)	266	53

Table 3 summarizes respondents’ attitudes toward water pollution. Over half (n = 263; 52%) of respondents had a positive attitude about measures to prevent water pollution. Most of them (77%) said that defecating near a water source can cause contamination, and that households are obliged to build their own latrines (99%). All participants (99%) stated that boiling water can eliminate bacteria.

**Table 3:** Respondents’ attitudes toward water pollution, Siguiri mining areas, Guinea, June 2023 (*n* = 501).

Variables	Frequency	Percentage
Is drinking water only good when you are ill?		
No	60	12
Yes/Do not know	441	88
Can drinking enough clean water prevent diarrheal disease?		
No/Do not know	16	3
Yes	485	97
Can defecating near a water source cause contamination?		
No/Do not know	114	32
Yes	387	77
Does boiling water before drinking help to eliminate pathogenic micro-organisms?		
No/Do not know	16	4
Yes	479	96
Do you think boiling water can remove the smell?		
No	228	46
Yes	273	54
Do you think boiling water can eliminate contaminants?		
No	12	2
Yes	489	98
Do you think boiling water removes chlorine?		
No	243	49
Yes	258	51
Do you think boiling water can eliminate bacteria?		
No	5	1
Yes	496	99
Do you think boiling water adds minerals?		
No	112	22
Yes	389	78
Are households obliged to build their own latrines?		
No/Do not know	4	1
Yes	495	99
Do households have to possess hand-washing facilities?		
No/Do not know	68	19
Yes	408	81
Total mean score (SD)	7,75 (± 1,21)	
Negative attitude (<8 points)	238	48
Positive attitude (≥8 points)	263	52

Table 4 shows respondents’ practices regarding water pollution. The majority (n = 453; 90%) used protected water sources for domestic activities and eight out of ten respondents treated drinking water at home. Nearly three quarters (73%) used an iron container to store water. All respondents (98%) said they used latrines. Providers who gave positive answers to these four (4) questions were classified as demonstrating good practices (*n* = 404; 81%) regarding water pollution.

**Table 4:** Respondents’ practices of water pollution, Siguiri mining areas, Guinea, June 2023 (*n* = 501).

Variables	Frequency	Percentage
How is your source of water supply?		
Unprotected (well/ river/ swamp)	48	10
Protected (mineral water/ pump/ borehole)	453	90
Do you treat drinking water at home?		
No	80	16
Yes	421	84
What type of storage container do you use?		
Iron container	368	73
Plastic container	135	27
Do you use latrines?		
No	8	2
Yes	493	98
Total Score	4	
Poor practice (<4 points)	97	19
Good practice (=4 points)	404	81

The bivariate analysis in Table 5 showed statistically significant associations between positive attitudes to water pollution in artisanal mining area and the variables age, occupation, and level of education.

**Table 5:** Factors associated with positive attitudes regarding water pollution in the mining areas of Siguiri, Guinea, June 2023 (*n* = 501).

Characteristic	ORb	95% CI		*p*-value	ORa	95% CI		*p*-value
		Lower	Upper			Lower	Upper	
Sex								
Female	Ref				Ref			
Male	0.77	0.51	1.15	0.199	0.73	0.47	1.14	0.169
Age_group								
<30	Ref				Ref			
30–45	0.73	0.51	1.07	0.106	0.78	0.53	1.15	0.205
>75	0.49	0.26	0.92	0.027	0.56	0.29	1.06	0.078
Marital status								
Married	Ref							
Single	1.16	0.81	1.68	0.413				
Occupation								
Mining worker	Ref				Ref			
Non-mining worker	1.45	1.01	2.07	0.042	1.33	0.91	1.95	0.143
Instruction level								
Unschooled	Ref				Ref			
Schooled	1.81	1.24	2.66	0.002	2.13	1.39	3.29	<0.001
Origin								
Foreigner	1.67	0.95	3.04	0.082	1.88	1.04	3.51	0.041
Guinean	Ref				Ref			
Life style								
Alcohol	Ref							
None	0.89	0.44	1.76	0.739				
Tobacco	0.72	0.35	1.49	0.380				
Religion								
Christian	Ref							
Fetishist	1.17	0.31	4.77	0.819				
Muslim	1.87	0.81	4.68	0.158				
Knowledge class								
Poor knowledge (= < 14 points)	Ref							
Good knowledge (>14 points)	0.76	0.53	1.08	0.122				

ORb, Odds Ratio brut; ORa, Odds Ratio ajusté; CI, Confidence Interval.

However, after adjustment by logistic regression, only level of education and origin were statistically significantly associated with positive attitudes toward water pollution in artisanal mining area. Compared to those without education, educated respondents were twice as likely to have positive attitudes towards water pollution (ORa: 2.13; 95% CI: 1.39; 3.29). In addition, being a foreigner was almost twice as likely to have positive attitudes compare to not being a foreigner (ORa: 1.88; 95% CI: 1.04; 3.51).

Table 6 shows the factors associated with good practices toward water pollution in the artisanal mining areas. The bivariate analysis showed statistically significant associations with the following variables: level of education, lifestyle, knowledge, and attitudes.

**Table 6:** Factors associated with good practices regarding water pollution in the mining areas of Siguiri, Guinea, June 2023 (*n* = 501).

Characteristic	ORb	95% CI		*p*-value	ORa	95% CI		*p*-value
		Lower	Upper			Lower	Upper	
Sex								
Male	Ref							
Female	1.00	0.59	1.64	0.995				
Age group								
<30	Ref							
30–45	0.97	0.61	1.56	0.913				
>45	1.44	0.64	3.66	0.408				
Marital status								
Married	Ref				Ref			
Single	1.38	0.86	2.24	0.188	1.84	1.10	3.14	0.022
Occupation								
Mining worker	Ref							
Non-mining worker	0.87	0.55	1.36	0.543				
Instruction level								
Unschooled	Ref				Ref			
Schooled	0.50	0.32	0.78	0.003	0.37	0.21	0.63	<0.001
Origin								
Guinean	Ref				Ref			
Foreigner	0.60	0.32	1.17	0.119	0.73	0.35	1.55	0.391
Life style								
Alcohol	Ref				Ref			
None	2.25	1.01	4.76	0.038	2.51	1.02	5.97	0.039
Tobacco	1.34	0.59	2.91	0.474	1.21	0.48	2.94	0.674
Religion								
Muslim	Ref							
Fetishist	1.98	0.36	37.0	0.521				
Christian	1.74	0.58	7.46	0.380				
Knowledge class								
Poor knowledge(= < 14 points)	Ref				Ref			
Good knowledge (>14 points)	1.72	1.10	2.70	0.018	2.51	1.47	4.36	<0.001

ORb, Odds Ratio brut; ORa, Odds Ratio ajusté; CI, Confidence Interval.

In the multivariate analysis, marital status, level of education, lifestyle and having good knowledge were statistically significantly associated with good practices toward water pollution. Being single (ORa: 1.84; 95% CI: 1.10; 3.14) and having good knowledge of water pollution (ORa: 2.51; 95% CI: 1.47; 4.36) increased the odds of having good practices regarding water pollution by almost two. However, having attended school (ORa: 0.37; 95% CI: 0.21; 0.63) was 63% less likely having a good practice towards water pollution in artisanal mining area. Compared with respondents who consumed tobacco and alcohol, those with no lifestyle (ORa: 2.51; 95% CI: 1.02; 5.97) had almost three times higher odds of having a good practice.

## 4 Discussion

The aim of this study was to assess the knowledge, attitudes and practices of the population in relation to water pollution in the artisanal mining areas of Siguiri, as well as the associated factors. The results highlighted average knowledge, positive attitudes and adequate practices in this population. Access to safe, available and accessible drinking water is essential for every individual (24).

A good knowledge of water pollution was observed in half of the respondents. Although the majority of respondents were aware that artisanal mining contaminates water sources with heavy metals and micro-organisms, significant gaps remained in understanding the specific risks associated with long-term exposure to heavy metals. A third of participants were unaware of the link between heavy metal ingestion and the development of cancers, a proven risk according to the scientific literature (25–27). This result may be attributed to the low level of education of the population in this region, where more than half of respondents have no formal education. It may also reflect the absence of targeted awareness campaigns on the environmental and health impacts of artisanal mining. A study of the impacts of artisanal gold mining in the Republic of Guinea revealed that ineffective enforcement of mining laws and a lack of environmental education contribute to an incomplete perception of the dangers associated with artisanal mining (22). This lack of awareness underlines the importance of targeted information campaigns on the specific dangers associated with heavy metal pollution in mining areas. Campaigns must include clear information on the sources of water pollution, health risks and accessible preventive measures. A study by Ab Razak et al. (28) in Malaysia on knowledge, attitudes, and practices concerning water pollution by metals reported that 80% of respondents were aware of heavy metal contamination in drinking water, and 70% knew they could be exposed to it. In another study, Berhe et al. (16) in Ethiopia found that 78.1% of adults knew about water safety, sanitation and hygiene, and that (82 to 98%) of participants recognized that unsafe drinking water can lead to diarrhea and other illnesses. Most respondents emphasized that ingesting germs could cause diarrheal diseases, which explains why eight out of ten respondents consider the presence of feces in nature to be a source of pollution. Consequently, the use of latrines is seen as essential and mandatory for every household.

Our results showed that around half the respondents have a positive attitude towards water pollution. Residents of artisanal mining areas are frequently confronted with the tangible consequences of this pollution, such as water-borne diseases, degraded water quality and limited access to clean water sources. These personal experiences can reinforce their awareness of the importance of protecting water resources. What’s more, in rural communities, water management is often seen as a collective responsibility, which can also foster positive attitudes in this regard. According to Ab Razak et al. (28), eight out of ten respondents have a positive attitude, as 59 % of participants had an intermediate level of education. Factors associated with a positive attitude in our study were respondents’ level of education and origin. Individuals with formal education were more likely to recognize the importance of water quality and to adopt environmental behaviors (29). This finding confirms that formal education is an essential lever for influencing behavior. Indeed, foreign respondents, who display more positive attitudes, have probably been exposed to better-regulated water management systems in the past. By reinforcing messages on the importance of water management, while taking into account local realities and traditional knowledge, it would be possible to broaden these favorable attitudes and transform them into sustainable behaviors.

In our study, the majority of respondents adopted good water management practices. This finding can be attributed to socio-economic conditions that influence behavior in the face of water pollution. In a context of limited resources, populations are often encouraged to adopt practices designed to protect their health and that of their families. Among these practices, we note in our study the preference for borehole water and sachet water over river or well water as the main sources of supply. The 2017 WHO/UNICEF survey (24) indicates that 48.7% of rural households in Guinea used at least one basic water supply service, reflecting increasing access to improved water sources. In addition, respondents in our study commonly adopted water treatment methods such as boiling and filtration (30, 31). This demonstrates a desire to reduce the risks associated with contamination and ensure safer water consumption (32).

Factors associated with the adoption of good practices include marital status, level of education, adequate knowledge and lifestyle. Respondents living alone may have more time and availability to inform themselves and implement preventive measures (33). However, it is paradoxical that the most educated individuals seem less inclined to follow traditional water pollution prevention practices. This may be because formal education does not sufficiently address local environmental issues, limiting specific awareness of water pollution. Although the knowledge acquired is supposed to translate into good practices, and almost all respondents reported the use of latrines, we found the presence of excrement in the environment. This highlights the gap between knowledge and action to prevent pollution (34). What’s more, people who do not have a lifestyle associated with tobacco and alcohol consumption may be more attentive to their overall health, and may be more inclined to adopt proactive behaviors, such as water treatment, to avoid water-borne illnesses. This observation could indicate greater awareness of healthy behaviors and the need to prevent water pollution.

To our knowledge, this is the first study conducted in Guinea, and possibly in Africa, to assess the knowledge, attitudes, and practices of local populations regarding water pollution in artisanal mining areas. It provides essential information to guide future interventions, improve awareness strategies, and promote sustainable behaviors through tailored educational programs. The findings support the development of public policies aimed at mitigating the negative impacts of artisanal mining, complemented by awareness programs focused on water management, a vital resource. They also contribute to strengthening local capacities while aligning with Sustainable Development Goal 6, which seeks universal access to clean and safely managed water. Moreover, this study offers an analytical framework applicable to similar contexts in sub-Saharan Africa, representing a significant step forward in addressing water pollution in mining regions. Despite these strengths, the study has certain limitations. First, as a cross-sectional study, its findings may not be generalizable and do not establish causal relationships between independent variables and the outcomes observed. Second, the reliance on self-reported data introduces the potential for response biases. Third, the study did not include qualitative data collection, which could have provided more in-depth insights from the respondents.

## 5 Conclusion

This study revealed moderate knowledge, generally positive attitudes, and largely adequate practices regarding water pollution in the artisanal mining areas of Siguiri. However, significant gaps remain, including a lack of awareness about the risks of prolonged exposure to heavy metals, disparities in attitudes, and inconsistencies in the implementation of optimal practices. To remedy these shortcomings, it is essential to enhance local community knowledge through targeted campaigns on the dangers of heavy metals and educational programs tailored to the cultural and environmental realities. Transforming this knowledge into sustainable attitudes and practices is essential for promoting concrete solutions, such as adopting water treatment technologies and strengthening community initiatives. These actions would directly contribute to public health and the protection of water resources in artisanal mining areas. Establishing a strategic partnership between the government, political actors, and stakeholders is also critical to consolidating progress and addressing persistent challenges. Furthermore, future research should delve deeper into the underlying mechanisms influencing knowledge, attitudes, and practices, using a mixed-methods approach that combines quantitative and qualitative methodologies.

## Data Availability

The raw data supporting the conclusions of this article will be made available by the authors, without undue reservation.
